# Supplementary material for: Obesity and its associated risk factors among school-aged children in Sharjah, UAE
Source: PLoS One. 2020 Jun 5;15(6):e0234244. doi: 10.1371/journal.pone.0234244 (PMC7274381; doi:10.1371/journal.pone.0234244)
Supplement: S1 File — (PDF) [file pone.0234244.s001.pdf]

College of Pharmacy

Consent Form

Dear Participant

You are invited to participate in a survey of a study titled:

"Obesity and its associated risk factors among school-aged children in Sharjah, UAE."

Purpose: The purpose of the study is to evaluate your child's daily habits, lifestyle and diet.

Description of procedure: The researchers will visit the school of your child to distribute a pre-piloted survey that has been estimated to be filled within 10-15 minutes. The aim of the survey will be carefully explained to you and researchers will be present to answer questions and queries.

The survey includes questions covering sociodemographic characteristics and others regarding various aspects related to the weight of your child. The collected data from surveys will be encoded and analyzed.

Your participation is completely voluntary, and your responses will be completely anonymous.

The data collected will be analyzed at the investigators level only. There are no consequences if you decide to withdraw from the study. - No benefit or risk will be gained from participation in this study. If you have any questions concerning your participation or the study protocol you can contact Dr. Suhail Al Amad, the head of the ethical committee through email: salamad@sharjah.ac.ae. or telephone number: 06-5057304 or Dr. Abduelmola R. Abduelkarem through email: aabdelkarim@sharjah.ac.ae or tel. 06-5057443. Your voluntarily participation is greatly appreciated.

You should read the participant information sheet and keep it for your record. Signing this form is considered your written consent and approval to voluntarily participate in the present study

Thank you for agreeing to participate in this study.

Name:

Signature: Date: / /2019
