# Supplementary material for: Obesity and its associated risk factors among school-aged children in Sharjah, UAE
Source: PLoS One. 2020 Jun 5;15(6):e0234244. doi: 10.1371/journal.pone.0234244 (PMC7274381; doi:10.1371/journal.pone.0234244)
Supplement: S2 File — (PDF) [file pone.0234244.s002.pdf]

College of Pharmacy

Dear Participant (Respected parents)

You are invited to participate in a survey of a study titled:

"Obesity and its associated risk factors among school-aged children in Sharjah, UAE"

Purpose: Purpose: The purpose of the study is to evaluate obesity and factors that contribute to overweight and obesity among school children.

Description of procedure: The researchers will distribute the survey that is estimated to take 10-15 minutes to fill it. The researcher will explain the purpose and nature of the investigation and answer any vague query.

-The survey includes questions covering sociodemographic characteristics and others regarding the lifestyle of children, their daily habits and diet. The collected data from surveys will be encoded and analyzed.

-Your participation is completely voluntary, and anonymous.

-The data collected will be analyzed at the investigators level only. There are no consequences if you decide to withdraw from the study. - No benefit or risk will be gained from participation in this study. If you have any questions concerning your participation or the study protocol you can contact Dr. Suhail Al Amad, Chair of Ethical Committee through email: salamad@sharjah.ac.ae. or telephone number: 06-5057304 or Prof. Abduelmola R Abduelkarem through email: aabdelkarim@sharjah.ac.ae or tel. 06-5057443. Your participation is greatly appreciated and please keep this information sheet for your record.
