# Supplementary material for: Obesity and its associated risk factors among school-aged children in Sharjah, UAE
Source: PLoS One. 2020 Jun 5;15(6):e0234244. doi: 10.1371/journal.pone.0234244 (PMC7274381; doi:10.1371/journal.pone.0234244)
Supplement: S3 File — (PDF) [file pone.0234244.s003.pdf]

جامعة الشارقة
كلية الصيدلة
قسم الصيدلة السريرية
مشروع تخرج طالبات السنة النهائية لكلية الصيدلة

أباءنا وأمهاتنا أولياء الأمور الكرام:
نحن طالبات السنة النهائية بكلية الصيدلة في جامعة الشارقة، نسعى لعمل مشروع التخرج بعنوان (سمنة الأطفال في الإمارات هل هي مشكلة ؟) تحت إشراف د.عبد المولى رجب عبد الكريم. ويدرس هذا المشروع ظاهرة السمنة لدى طلاب/طالبات بعض مدارس دولة الإمارات العربية المتحدة الذين يتراوح اعمارهم من (6-11) . لذا نود منكم يد العون ومشاركتنا ذلك بمساعدة أبناءكم و بناتكم الأعزاء في ملئ هذا الاستبيان وارساله معهم في اليوم التالي للمدرسة حيث ستقوم ادارة المدرسة بجمعه منهم، علما بأن مشاركتكم ستساهم بعون الله في دعم مسيرة البحث العلمي في هذا المجال والذي يهدف لإبعاد شبح الأمراض المزمنة (المتعلقة بالسمنة) عن أولادنا مستقبلا، وذلك بنشر الوعي الصحي بينهم من خلال مدارسهم، علما بأن جميع البيانات المذكورة ستعامل بالسرية التامة.
(راجين منكم تدوين المعلومات بدقة نظرا لأهمية البحث وجدية الأمر)

الاستبيان

نرجو من سيادتكم وضع إشارة √ على الإجابة المناسبة أو كتابة الإجابة على الفراغ المناسب:

<u>بيانات الطالب/ة:</u>
<u>*الأسئلة من 4 الى 6 سيتم تعبئتهم من قبل الباحثين*</u>

1. الجنس : ☐ انثى ☐ ذكر
2. العمر :____________ الصف:____________
3. الجنسية : ☐عربي ☐ غير عربي
4. الوزن : ____________
5. الطول: ____________
6. مؤشر كتلة الجسم :

☐ الوزن الناقص ☐ الوزن الطبيعي ☐ الوزن الزائد ☐ السمنة

التغذية و اللياقة البدنية للطالب/ة:

7. هل يوجود احد في العائلة مصاب بالسمنة : ☐ نعم ☐ لا

8. في المدرسة , عادةً :

☐ يحضر طفلك الغداء من المنزل
☐ يشتري طفلك وجبته من الكافتريا
☐ يكتفي طفلك بوجبة خفيفة
☐ يتخطى طفلك وجبة الغداء

9. كم وجبة يأكل طفلك في اليوم :
☐ 1 ☐ 2-5 ☐ أكثرمن 5

10. المدة الفاصلة بين الوجبة الاخيرة (العشاء) و النوم ؟
☐ 0- 30 دقيقة
☐ دقيقة 30- 1 ساعة
☐ 1 ساعة – 2 ساعتين
☐ أكثر من 2 ساعتين

11. عدد المرات التي يأكل فيها طفلك الوجبات السريعة ؟
☐ دائماً (كل يوم او في أغلب الايام)
☐ عادةً (مرة في الاسبوع)
☐ احياناً (مرة في الشهر)
☐ نادراً

12. عدد المرات التي يأكل فيها طفلك الحلوى ؟
☐ دائماً (كل يوم او في أغلب الايام)
☐ عادةً (مرة في الاسبوع)
☐ احياناً (مرة في الشهر)
☐ نادراً

13. كم وجبة من وجبات طفلك اليومية تحتوي على طعام صحي(الفواكه و الخضروات و الخ) ؟
☐ 0 ☐ 1 ☐ 2 ☐ 3 ☐ 4 أو أكثر

14. ما طبيعة الطعام الذي يأكله طفلك ؟
☐ غالبا مسلوق ☐ غالبا مقلي ☐ غالبا مشوي ☐ منوع

15. معظم طعام طفلك مكون من :
☐ كربوهيدرات ☐ سكريات ☐ دهون ☐ بروتينات ☐ لا اعلم

16. كم من الوقت يستغرق طفلك في مشاهدة التلفاز ؟
- ☐ 30 دقيقية
- ☐ 30 دقيقة - 1 ساعة
- ☐ 1 ساعة – 2 ساعتين
- ☐ أكثر من ساعتين

17. كم من الوقت يستغرق طفلك في استخدام (الهاتف المحمول , التابلت , الحاسوب) ؟
- ☐ 30 دقيقية
- ☐ 30 دقيقة - 1 ساعة
- ☐ 1 ساعة – 2 ساعتين
- ☐ أكثر من ساعتين

18. هل يعاني طفلك من اي امراض ؟
- ☐ نعم, إذكر/ي ________________
- ☐ لا

19. هل يأخذ طفلك اي ادوية ؟
- ☐ نعم, إذكر/ي ________________
- ☐ لا

20. هل يمارس طفلك الرياضة او اي من النشاطات الخارجية ؟
- ☐ دائماً (كل يوم او ي أغلب الايام)
- ☐ عادةً (مرة في الاسبوع)
- ☐ احياناً (مرة في الشهر)
- ☐ نادراً

ختاماً نشكر لكم حسن تعاونكم، راجين من الله تعالى كل التوفيق.
