# Supplementary material for: Obesity and its associated risk factors among school-aged children in Sharjah, UAE
Source: PLoS One. 2020 Jun 5;15(6):e0234244. doi: 10.1371/journal.pone.0234244 (PMC7274381; doi:10.1371/journal.pone.0234244)
Supplement: S4 File — (PDF) [file pone.0234244.s004.pdf]

**College of Pharmacy**

**Department of Pharmacy Practice & Pharmacotherapeutics**

**Title: <u>Obesity and its associated risk factors among school-aged children in Sharjah, UAE</u>**

**Dear respected parents:**

**This project aims at investigation the prevalence of overweight and obesity among school children. Your voluntary participation in filling the questionnaire will help us in studying the problem of obesity and pinpointing the risk factors contributing to the problem. Our trained researchers with your permission will non-invasively measure some parameters of the children in school under the supervision of school teachers. Your identity is not revealed at any stage of the study and you are free to withdraw at any stage of the study. Your help and cooperation by willingly participating would be greatly appreciated**.

## SECTION 1: SOCIO-DEMOGRAPHIC AND CLINICAL CHARACTERISTICS

1. Gender: ☐ Male ☐ Female

2. Age: _______

3. Nationality: ☐ Arab ☐ Non-Arab

4. Weight: ______

5. Height: ______

6. BMI-P: ______

   ☐ Underweight ☐ Normal ☐ Overweight ☐ Obese

7. Family history of obesity: ☐ Yes ☐ No

## SECTION 2: CHILD HABBITS AND LIFESTYLE

8. At school, you usually:

☐ Bring your lunch from home.

☐ Buy a meal from the cafeteria.

☐ Just eat snacks.

☐ I skip lunch.

9. How many meals do you eat during the day?

☐ 1 ☐ 2-5 ☐ More than 5

10. How much time do you leave between your last meal (dinner) and bedtime?

0-30 minutes

☐ 30 minutes – 1 hour

☐ 1 hour to 2 hours

☐ More than 2 hours

11. How often do you eat fast-food?

☐ Always (Everyday or almost everyday)

☐ Often (A few times a week)

☐ Sometimes (About once a week)

☐ Rare

12. How often do you eat candy?

☐ Always (Everyday or almost everyday)

☐ Often (A few times a week)

☐ Sometimes (About once a week)

☐ Rare

13. How many of your meals include healthy food (Fruits/Vegetables/etc.)?

☐ 0 ☐ 1 ☐ 2 ☐ 3 ☐ 4 or more

14. How much time do you spend watching TV?

- ☐ 30 minutes
- ☐ 30 minutes to 1 hour
- ☐ 1 hour to 2 hours
- ☐ More than 2 hours

15. How much time you spend on your (phone, tablet, computer):

- ☐ 30 minutes
- ☐ 30 minutes to 1 hour
- ☐ 1 hour to 2 hours
- ☐ More than 2 hours

16. Do you suffer from any disease?

- ☐ No
- ☐ Yes, mention please _______________

17. Are you on any medication or supplements?

- ☐ No
- ☐ Yes, mention please________________

18. How often do you workout or go on for outside activity?

- ☐ Always (everyday or almost everyday)
- ☐ Often (once a week)
- ☐ Sometimes (once a month)
- ☐ Rare
