# Supplementary material for: Obesity and its associated risk factors among school-aged children in Sharjah, UAE
Source: PLoS One. 2020 Jun 5;15(6):e0234244. doi: 10.1371/journal.pone.0234244 (PMC7274381; doi:10.1371/journal.pone.0234244)
Supplement: Attachment — Submitted filename: comments to authors.docx [file pone.0234244.s005.docx]

*Comments to the Author*

*This Study aimed to assess “obesity and its associated risk factors among school-aged children in sharjah”*

1- *In the introduction section the authors refer to the prevalence of childhood overweight and obesity in the US and worldwide, but they did not provide any description about the prevalence of childhood overweight and obesity in the UAE. Please add this data in the introduction.*
2- *Anthropometric measurements: Quality assurance for anthropocentric measurements should be described in details.*
3- *Is the questionnaire of dietary habit reliable or valid for this population study? Please provide more information about reliability and validity of this questionnaire.*
4- *The author did not provide any information about the physical activity in children. Please add this information in the methods section (questionnaire development section).*
5- *Based on method section you select population between native and non-native students, but in the results you did not refer to this. How many of the subjects were non-native? Are there any differences in the prevalence of childhood overweight and obesity between the native and non-native students?*
6- *What is the definition of physical inactivity that you reported in the result section?*
7- *In the statistical analysis the author used the chi-square test to ascertain the association between the dependent and independent variables, how you can use Chi-square test for ascertain an association? Please check the type of statistical tests with a statistician.*
8- *Table section: The author must mention that the type of statistical tests was used for assessing the difference of various socio-demographic, lifestyle, anthropometric, in across BMI percentile.*
9- *In the table you did not refer that what type of data was used, number or percent?*
