# Supplementary material for: Obesity and its associated risk factors among school-aged children in Sharjah, UAE
Source: PLoS One. 2020 Jun 5;15(6):e0234244. doi: 10.1371/journal.pone.0234244 (PMC7274381; doi:10.1371/journal.pone.0234244)
Supplement: Attachment — Submitted filename: 3-Our Response to Reviewers Comments.docx [file pone.0234244.s006.docx]

**Our Responses to Reviewers Comments:**

| **Comments: Reviewer 1** | **Our Responses** |
| --- | --- |
| 1- In the introduction section the authors refer to the prevalence of childhood overweight and obesity in the US and worldwide, but they did not provide any description about the prevalence of childhood overweight and obesity in the UAE. Please add this data in the introduction. | Lines 61-67:In UAE, the prevalence of overweight and obesity varied across age, where below 9 years of age both sexes were below the international standards with an increase overweight and obesity occurred among both genders of 9 to 18 years [AL-HADDAD et al., 2005]. A more recent study demonstrated that in UAE, obesity starts in toddlers and progresses linearly with age and pointed out an alarming high prevalence of extreme obesity, especially among boys [AlBlooshi , S. Shaban , M. AlTunaiji3 , N. Fares , L. AlShehhi , H. AlShehhi , A. AlMazrouei and A. -K. Souid. Increasing obesity rates in school children in United Arab Emirates. Obesity Science & Practice, 2016, doi: 10.1002/osp4.37]. The sequence of references was readjusted accordingly. |
| 2- Anthropometric measurements: Quality assurance for anthropocentric measurements should be described in details. | The authors are thankful for this important suggestion.  -Lines 151-188: Details of [anthropometric](https://www.sciencedirect.com/topics/medicine-and-dentistry/anthropometry) measurements are added.  -Lines 151-188: Detailed description of the quality assurance of [anthropometric](https://www.sciencedirect.com/topics/medicine-and-dentistry/anthropometry) measurements is added.  Information pertaining to quality assurance is updated. Trained researchers measured student’s height and weight. The students were instructed to stand straight with their heads, backs and buttocks vertically aligned to the height gage, and then their heights were taken and rounded to the nearest 0.5 cm. Simultaneously the students’ weights were recorded from the digital screen and rounded to the nearest 0.5 kg. |
| 3- Is the questionnaire of dietary habit reliable or valid for this population study? Please provide more information about reliability and validity of this questionnaire. | Lines 112-115: The questionnaire of dietary habit is reliable as it is to be filled in by parents of the school children. It was pre-piloted for face validity by distributing it to 12 national and non-national parents of school children of the targeted age and their comments and recommendation were taken into consideration in the final version of the survey. |
| 4- The author did not provide any information about the physical activity in children. Please add this information in the methods section (questionnaire development section). | Lines 124-127: In this context we defined physical activity as any indoor or outdoor sport activity not including the use of smart devices or watching TV. On the hand physical inactivity is defined as sedentary pleasurable activities such as watching cartoons on TV or using smart devices for gaming. |
| 5- Based on method section you select population between native and non-native students, but in the results you did not refer to this. How many of the subjects were non-native? Are there any differences in the prevalence of childhood overweight and obesity between the native and non-native students? | In the method section, selection of population was general without any attempt to trace child’s nationality as at the targeted age group most, if not all, non-national children were born and raised in UAE. Therefore, no attempts were made to categorize children and study the prevalence of overweight and obesity in each category. On the basis of this, the statement pointing at national and non-national students was deleted from the method section in the abstract. |
| 6- What is the definition of physical inactivity that you reported in the result section? | Lines 126-127: The definition of physical inactivity was included in the questionnaire development section. (On the other hand physical inactivity is defined as sedentary pleasurable activities such as watching cartoons on TV or using smart devices for gaming.) |
| 7- In the statistical analysis the author used the chi-square test to ascertain the association between the dependent and independent variables, how you can use Chi-square test for ascertain an association? Please check the type of statistical tests with a statistician. | The chi-square test for association (contingency) is a standard measure for association between two categorical variables. The chi-square test, unlike Pearson's correlation coefficient or Spearman rho, is a measure of the significance of the association. It has been used in other similar studies where the relationship between each factor of interest and children’s weigh status and others was explored by (%) or χ^2^ test for categorical variables. |
| 8- Table section: The author must mention that the type of statistical tests was used for assessing the difference of various socio-demographic, lifestyle, anthropometric, in across BMI percentile. | The test used is mentioned in table 4, lines 245 & 255 and in table 5, line 265. |
| 9- In the table you did not refer that what type of data was used, number or percent? | In all tables the data was readjusted to denote Frequency (%) |

| **Comments: Reviewer 2** | **Our Response** |
| --- | --- |
| **Abstract**: 1. Line 36: Should read “…and determine” rather than determining  2. Line 38: insert “was” before the word based  3. Line 43: Recommend person-first language. Would recommend from “were obese or overweight” to “had obesity or overweight.” | Line 37: Corrected. “…and determine the influence.”  Line 38: Corrected. “The study was based.’  Line 43: Corrected. “ ..children had obesity” |
| **Introduction**: 4. Line 65-66: The consumption of high calories food with no or limited physical activity is the main contributor to childhood obesity. This statement needs a citation.  5. Line 73: Run on sentence, needs appropriate punctuation.  6. Line 80: Two strategies can be followed including; the primary prevention of overweight or obesity itself and second is the prevention of weight regain after weight loss, plus prevention of weight gain in individuals unable to lose weight [5]. This statement is confusing. Initially talks about two strategies, but introduces a third.  7. Line 100: Add “the” before the word questionnaire. | Lines 73-75: Citation was made. “The consumption of high-calories food with no or limited physical activity is the main contributor to childhood obesity [8]. The sequence of references was readjusted accordingly.  The authors are thankful for the suggestion. Reference updated. Statement amended to both over-consumption of calories and reduced physical activity are involved in childhood obesity.  Line 73:Dehghan M, Akhtar-Danesh N, Merchant A. Childhood obesity, prevalence and prevention. Nutr J. 2005;4(1).DOI:10.1186/1475-2891-4-24  Line 73: Additionally, psychological factors such as familial stress, anxiety, and social isolation may contribute to childhood obesity.  Lines 88: Changed to: “Strategies that can be followed including; the primary prevention of overweight or obesity itself, and the second is the prevention of weight regain after weight loss plus prevention of weight gain in individuals who are unable to lose weight [7].  Line 102: ”the” was added: “the development of the questionnaire..” |
| **Methods**: 8. The survey question asking the Nature of food consumed to parents is very confusing. Why were fruit and vegetables not included as an answer choice for the question: predominant food in child’s diet? What does mixed mean – 2 categories, 3 categories? Would be better to have had families give percentages. Was fruit included in carbohydrate? Was the any ascertainment of baseline knowledge level – were families clear on what food groups fell into each of these categories? | The fruit and vegetables were included in the question. For each category examples were given and answers were noted. In our study mixed refers to combination of 3 categories of food. Fruit was included in carbohydrates. The same had been updated in table 2 (line 221). |
| 9. I am concerned about selection bias. The parents/child that returned the survey could be inherently different that the ones that didn’t. Did you have any data to say that your sample population is representative? Do you have any data on the parents/children that chose not to participate or the data on the schools as a whole? | We agree that the parents are inherently different than the ones who did not return the survey paper. Our study outcome was independent of ethnicity. From the list obtained from the Ministry of education we found that 8 Schools in Sharjah had a total of 65473 students. <https://data.bayanat.ae/en_GB/dataset/private-education-students-by-education-zone-level-and-gender/resource/0190ac38-ab63-49de-9ba3-0105760f16d9>. We calculated our sample size based on this number. Reference had been updated. We enrolled the participants based on the return of questionnaires. Those who did not return the questionnaires were not enrolled in the study |
| Study Population 10. How many schools were participants recruited from? Were the schools the same in terms of demographics of students?  11. Were the surveys sent to every child in a specific grade. Need to expand on how the sample was put together in more detail. | The participants were recruited from 8 schools. The schools were the same in terms of demographics. Information can be found in line 136.  In the study, within each selected school, pupils from Grade I to V were offered the opportunity to voluntarily and anonymously participate. Information updated in line 138-140. |
| **Results**: 12. Line 153: Person first language recommended. (E.g. Had an obese BMI, rather than were obese.)  13. Descriptive characteristics should be presented by BMI category.  14. It is unclear how disease status and medication use were related to the aims of the study | Lines 206-207: corrected.” A total of 487 (59.4%) children showed normal range of BMI whereas 95 (14%) and 96 (14.2%) had an obese BMI and overweight respectively.  Descriptive characteristics are now presented by BMI category.  Disease status and medication use were part of health related characteristics. It is removed from the aims of the study. |
